# A Comparison of Flavorless Electronic Cigarette-Generated Aerosol and Conventional Cigarette Smoke on the Survival and Growth of Common Oral Commensal *Streptococci*

**DOI:** 10.3390/ijerph16101669

**Published:** 2019-05-14

**Authors:** Giancarlo A. Cuadra, Maxwell T. Smith, John M. Nelson, Emma K. Loh, Dominic L. Palazzolo

**Affiliations:** 1 Department of Biology, Muhlenberg College, Allentown, PA 18104, USA; giancarlocuadra@muhlenberg.edu (G.A.C.); el251159@muhlenberg.edu (E.K.L.); 2 Department of Medical Laboratory Science, School of Allied Health Sciences, Lincoln Memorial University, Harrogate, TN 37752, USA; maxwellsmith1572@gmail.com; 3 Department of Biology, School of Mathematics and Sciences, Lincoln Memorial University, Harrogate, TN 37752, USA; john.nelson@LMUnet.edu; 4 Department of Physiology, DeBusk College of Osteopathic Medicine, Lincoln Memorial University, Harrogate, TN 37752, USA

**Keywords:** ECIG, E-liquid, vaping, smoking, aerosol, *streptococci*, oral commensal bacteria

## Abstract

*Background:* The use of electronic cigarettes (ECIG) has become very common. Consequently, critical analysis of the biological effects of ECIG aerosol deserves attention. Flavorless ECIG aerosol is known to comprise fewer harmful constituents than cigarette smoke. Therefore, we hypothesize that aerosol has less immediate effect on the viability of oral commensal *streptococci* than smoke. *Methods:* Survival and growth of four strains of commensal *streptococci* were measured after exposure to flavorless ECIG aerosol ± nicotine and smoke. Peristaltic pumps were used to transport aerosol or smoke into chambers containing recently seeded colony-forming units (CFUs) of the oral commensal *streptococci* on agar plates. Bacterial survival and growth, based on colony counts and sizes, were determined 24 h post-exposure. Additionally, aerosol or smoke were delivered into chambers containing pre-adhered *streptococci* to plastic coverslips and biofilm formation was determined 24 h post-exposure via scanning electron microscopy. *Results:* The results suggest that flavorless aerosol ± nicotine has a modest effect on bacterial growth both as colonies on agar and as biofilms. In contrast, smoke dramatically decreased bacterial survival and growth in all parameters measured. *Conclusion:* Unlike cigarette smoke, flavorless ECIG aerosol has only a small effect on the survival and growth of oral commensal *streptococci*.

## 1. Introduction

The use of electronic cigarettes (ECIG), referred to as vaping, has gained immense popularity in recent times [1]. Cigarette smoke is known to contain thousands of detrimental compounds, but the constituents of flavorless ECIG aerosol are few. In general, ECIG-liquid (E-liquid) consists of propylene glycol and/or vegetable glycerin, nicotine ranging from 0 to >24 mg/mL and a variety of flavors [2]. While vaping on ECIG devices is commonplace, as of yet there is no clear evidence of the potential issues its usage could cause. For this reason, the physiological effects of ECIG aerosol should be seriously investigated.

Currently, there are few studies regarding effects of ECIG-generated aerosol on physiological systems as compared to cigarette smoke. A few reports claim that ECIG use is as dangerous (or more dangerous) than traditional smoking [3,4,5]. E-liquid flavorings have also recently been reported to induce inflammatory and oxidative responses in human monocytic cell lines [6]. Similarly, various flavored E-liquids have a toxic effect on stem cells and terminally differentiated cell lines [7]. Moreover, human bronchial epithelial as well as oral epithelial cell lines exposed to ECIG-generated aerosol with flavorings increased pro-inflammatory cytokine production and caused other adverse effects on the biology of these cell models [8,9,10]. All these studies indicate that it is the flavorings, and not the base humectants (i.e., propylene glycol and/or vegetable glycerin) that is detrimental to exposed tissues and consequently deserves more attention to better inform the public, especially since many young adults are enticed by the myriad of flavors. On the other hand, the purpose of this study is to investigate the effects of a flavorless ECIG-generated aerosol on the survival and growth of several strains of oral commensal *streptococci*, important bacteria needed for the formation of biofilms. To our knowledge, there are no studies that have addressed the effects of flavorless ECIG aerosol on any oral bacteria. Analysis of the effects of a flavorless aerosol on the oral microbiome is important because it will allow for baseline quantification of streptococcal survival and growth. This information will eventually be useful in teasing out the absolute effect of individual flavors from the effects of the base humectants. For example, is the effect of the aerosol due to the vaporized flavors, the vaporized humectants or both? Thus, the results of this investigation will ultimately serve as a preamble for future studies related to the effects of flavored ECIG aerosol on the oral microbiome.

The oral cavity contains a vast diversity of commensal, opportunistic and sometimes pathogenic bacteria. The most common types of commensal bacteria are *streptococci* [11,12], which are found in individuals at any level of oral health and disease [13,14]. Among these bacteria, some of the most common species are *Streptococcus gordonii*, *Streptococcus intermedius*, *Streptococcus mitis* and *Streptococcus oralis* [15,16,17]. All four of these species are crucial in the development of oral biofilms on both soft and hard surfaces within the mouth [13,18,19]. These species are considered commensal early colonizers [20,21,22]. All four species are beneficial to the host oral cavity in the context of their interactions with pathogenic species related both to caries and periodontal disease [23,24,25,26,27,28]. By extension, since oral health and overall general health are directly correlated, any disruption to the bacterial flora within the oral cavity could lead to systemic diseases, especially certain types of cardiovascular disease [29]. For this reason, it is important to examine how vaping affects the oral commensal microbiota.

Smoking has been reported to be a leading risk factor for caries and periodontal disease [30,31,32,33] and is known to affect the subgingival oral microbiome in situ considerably [34]. No studies (to our knowledge) are available to show how ECIG aerosol specifically affects oral commensal *streptococci* known to provide a protective barrier against external insults. Since *S. gordonii*, *S. intermedius*, *S. mitis* and *S. oralis* are crucial in the development of oral biofilms on both soft and hard surfaces within the mouth, the specific aim of this work is to test the impact of flavorless ECIG aerosol and compare it to conventional cigarette smoke on the survival and growth of oral commensal streptococci.

## 2. Materials and Methods

### 2.1. Reagents and Supplies

All reagents and supplies for conducting these investigations were purchased from Thermo Fisher Scientific (Waltham, MA, USA) unless otherwise noted.

### 2.2. Bacterial Strains

*S. gordonii* DL1, *S. intermedius* 0809, *S. mitis* UF2 and *S. oralis* SK139 were kindly provided by Dr. Robert Burne from the University of Florida. All strains were grown in brain heart infusion (BHI) broth with 5 μg/mL hemin or BHI agar at 37 °C and 5% CO_2_. Bacterial stocks were stored at −80 °C.

### 2.3. E-Liquid

E-liquid was composed of 50% propylene glycol and 50% vegetable glycerin (i.e., glycerol) with or without (±) 20 mg/mL of (S)-(-)-nicotine (Alpha Aesar, Tewksbury, MA, USA). No flavors were added. This nicotine concentration on a per cigarette equivalent is higher than the typical concentration of nicotine in a tobacco cigarette [35], but is comparable to the high end nicotine concentration found in a number of commercially available E-liquids.

### 2.4. Exposure Apparatus

**Figure 1:**
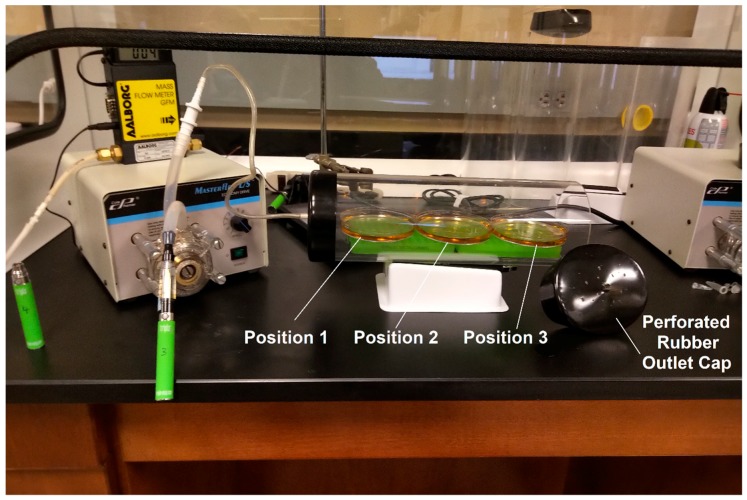
Exposure apparatus for flavorless electronic cigarettes (ECIG) aerosol and cigarette smoke. Peristaltic pumps, tubing and acrylic chamber containing three plates numbered 1, 2 and 3 according to their relative position with respect to the aerosol or smoke source (tubing connection to chamber). Mass flow meter (small yellow and black machine) was used to calibrate the flow of materials at 400 mL/min. The rubber outlet cap is removed to show perforations. All materials are shown inside a Purair ductless fume hood.

Bacterial samples were exposed to either air, flavorless ECIG aerosol ± 20 mg/mL nicotine or cigarette smoke following already established protocols [36,37]. Briefly, Cole-Palmer Master Flex L/S peristaltic pumps (Vernon Hills, IL) and tubing were used to simulate puffing and transport air, smoke or aerosol into an acrylic chamber, as shown in Figure 1. Peristalsis and flow rates were adjusted to 400 mL/min or 33.3 mL in five seconds as indicated in Table 1. Puffing was conducted at 5 s (pumps active) followed by a 10-second rest period (pumps inactive). The puffing protocol consisted of 0, 10, 25, 50 and 75 puffs. Values of total nicotine exposure in the acrylic chamber are shown in Table 1. All pump-puffing experiments were conducted within a P20 Purair ductless fume hood (Airscience, Fort Myers, FL, USA) with a high efficiency particulate air (HEPA) filter.

### 2.5. Distribution of Aerosol and Smoke in the Exposure Chamber

Three 100 mm plates containing 10 mL of BHI broth were placed in the exposure chamber. According to Figure 1, the plate in position 1 is the closest to the source of aerosol or smoke and the plate in position 3 is the farthest away. The rubber cap closest to position 3 in the chamber was perforated so as to not completely cut off ventilation, and thus limiting the buildup of excess aerosol or smoke. BHI broth in three plates was exposed to flavorless ECIG aerosol with 20 mg/mL nicotine and cigarette smoke for a total of 10, 25, 50 and 75 puffs following above protocols. Concentrations of nicotine in the BHI broth of plate 1, plate 2 and plate 3 (Figure 1) were evaluated by high-performance liquid chromatography (HPLC).

### 2.6. High-Performance Liquid Chromatography (HPLC) Determination of Nicotine

Standard solutions of 99% (S)-(-)-nicotine, were prepared in BHI broth at concentrations of 0.4, 0.2 and 0.1 mg/mL. Standards and samples of BHI exposed to 10, 25, 50 or 75 puffs of flavorless ECIG aerosol with nicotine or conventional cigarette smoke were analyzed by HPLC coupled with photodiode array detection as previously described [38,39]. A Shimadzu HPLC system (Columbia, MD) was used to quantitate nicotine and included the following: a photodiode array detector (SPD-M20A), dual pumps (LC-20AT), a column oven (CTO-20A), an in-line membrane degasser (DGU-20A3R) and a Rheodyne 7725I manual injector with 20 µL loop (40 µL injection volume). Nicotine was separated on a Phenomenex (Torrance, CA, USA) 15-cm, Kinetex^®^ 5 µm reversed phase C-18 column preceded by a Phenomenex Security Guard. Column temperature was maintained at 35 °C. Nicotine was detected at ultraviolet (UV) wavelengths between 230 and 300 nm and quantifications were carried out at 260 nm. The mobile phase was delivered at a rate of 1 mL/minute in gradient fashion where mobile phase A consisted of 10% acetonitrile in 20 mM ammonium formate adjusted to pH 8.5 with 50% ammonium hydroxide and mobile phase B consisted of 100% acetonitrile. Mobile phase A decreased from 100% to 80% from 0 to 10 min, decreased from 80% to 20% from 10 to 20 min, increased from 20% to 100% from 20 to 21 min and remained at 100% till the end of the run time at 30 min. Mobile phase B increased from 0% to 20% from 0 to 10 min, increased from 20% to 80% from 10 to 20 min, decreased from 80% to 0% from 20 to 21 min and remained at 0% till the end of the run time at 30 min. The nicotine standard curve was linear (R^2^ = 0.9998) and nicotine eluted at a retention time of 10.5 min. Chromatographic parameters were PC-controlled using a Shimadzu Lab Solutions work station (Columbia MD).

### 2.7. Colony-Forming Units (CFUs)

Starter overnight cultures of all four strains of bacteria were adjusted to optical density (OD) of 1 at 595 nm. For all species tested, an OD of 1.0 yields a range of 2 to 4 × 10^9^ CFU/mL. Then, bacteria were serially diluted (1:10) to numbers permissible for CFU counting and 20 μL of each species were plated in triplicate on BHI agar plates. As soon as the 20 µL volume dried into the agar, bacteria were exposed uncovered to air, flavorless ECIG aerosol (± 20 mg/mL nicotine) or cigarette smoke for up to 75 puffs. Following exposures, plates were incubated at 37 °C and 5% CO_2_ for 24 h. The next day, colonies were digitally photographed using a Moticam 1080 HDMI and USB camera (Motic^®^, Richmond, British Columbia, Canada) attached to a Fisher brand stereomicroscope and counted. Average colony sizes (as indexed by the area of individual colonies) were determined using the Moticam supplied on-board camera software (Motic^®^, Richmond, BC, Canada).

### 2.8. Biofilm Biomass

Starter overnight cultures of bacteria were adjusted to OD 595 nm of 1.00. After adjustment, 100 μL of each culture was seeded separately on sterile and untreated plastic coverslips (13 mm diameter) 12-well plates. Bacteria were allowed to adhere to the surface of the coverslips without agitation for 1 h at 37 °C, 5% CO_2_ and the excess unbound bacteria were washed 3 times with 0.5 mL phosphate-buffered saline (PBS). Excess liquid on the coverslips was removed and the 12-well plates containing the coverslips were exposed uncovered to 75 puffs air, flavorless ECIG-generated (±20 mg/mL nicotine) or cigarette smoke. Following exposure, 1 mL of 50% BHI broth (v/v in sterile water) was added to each well of the 12-well plate ensuring that exposed coverslips were completely submerged. Exposed bacteria were subsequently incubated without agitation and without media exchange for 24 h at 37 °C, 5% CO_2_ to allow for biofilm growth on the coverslips. At the end of the 24-h incubation period, BHI broth was removed from the wells and the coverslips were washed 3 times with 1 mL PBS to remove excess unbound bacteria. Biofilms were fixed with 1 mL of 4% formaldehyde for at least 30 min. Coverslips were then processed for scanning electron microscope (SEM) imaging (described below).

### 2.9. Biofilm Processing for Scanning Electron Microscope (SEM) Imaging

The 4% formaldehyde was removed from each well and each coverslip was rinsed two times with 1 mL of deionized water. The biofilms on the coverslips were then dehydrated using an increasing alcohol gradient (i.e., 30 min in each of 50, 70, 90 and 100% ethanol) followed by chemical drying with 98% hexamethyldisilizane for 30 min. The coverslips with attached biofilms were then removed from the 12-well plates and air dried for 5 to 10 min before mounting on to 13 mm aluminum pin-type stubs (Structure Probe, Inc. (SPI), West Chester, PA, USA). Conductive, 12 mm diameter, double-sided carbon-impregnated adhesive disks (SPI) were used to adhere the coverslips to the stubs and 1 to 2 h was allowed for complete adherence. In the mounting process, extreme care was used to ensure the side of the coverslip with the bacterial biofilm was facing up and not disrupted. The mounted bacterial biofilms were then sputter coated using a Hummer IV-A sputtering system (Anatech Ltd., Alexandria, VA, USA) and plated with 300Å of 1:1 gold:palladium. SEM images of biofilms grown on coverslips were taken with a TOPCON ABT-60 microscope at an acceleration voltage of 15 kV and a magnification of 450×.

### 2.10. Statistical Analysis

Mean and standard error of the mean (SE) were calculated for nicotine in BHI broth. One-way analysis of variance (ANOVA) followed by Newman–Keuls multiple comparison test analysis was used to determine differences in nicotine concentrations between plate positions 1, 2 and 3 after 10, 25, 50 and 75 puffs of flavorless ECIG aerosol with 20 mg/mL nicotine or conventional cigarette smoke. CFUs were visually counted and the average of three largest colonies in each quadrant of an agar plate were used to determine mean colony size for all bacteria at every exposure. Mean and standard error (SE) were calculated for CFU counts and colony size. Statistical variance between groups was determined using two-way ANOVA, followed by Bonferroni post hoc analysis. Differences were considered statistically significant when *p* < 0.01.

## 3. Results

### 3.1. Distribution of Aerosol and Smoke in the Exposure Chamber

**Figure 2:**
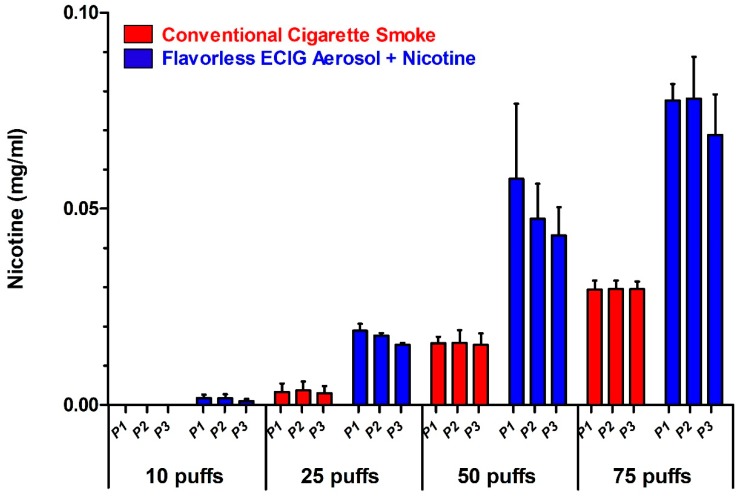
Levels of nicotine in three plates inside the exposure chamber. Nicotine concentrations (mg/mL) in three plates of brain heart infusion (BHI) broth (i.e., P1, P2 and P3) set up in tandem inside an exposure chamber after 10, 25, 50 and 75 puffs of flavorless ECIG aerosol containing 20 mg/mL nicotine or conventional cigarette smoke. Each data point is the Mean ± SE of 3 replicate exposures over 3 separate occasions (*n* = 3).

Figure 1 shows the setup of three plates in tandem inside the acrylic chamber. The BHI broth in all three plate positions received comparable amounts of nicotine (Figure 2, *p* > 0.05). The results also show that the amount of nicotine, regardless of source, increases in a dose-dependent manner in all three plates. Lastly, the results also show the projected results of higher levels of nicotine from flavorless ECIG aerosol compared to cigarette smoke (Figure 2) and agree with the expected values shown in Table 1. These data indicate that plate position is not a confounding factor in the results and interpretation of the following experiments.

### 3.2. Effects of Electronic Cigarette (ECIG)-Generated Aerosol and Smoke on CFU Counts

**Figure 3:**
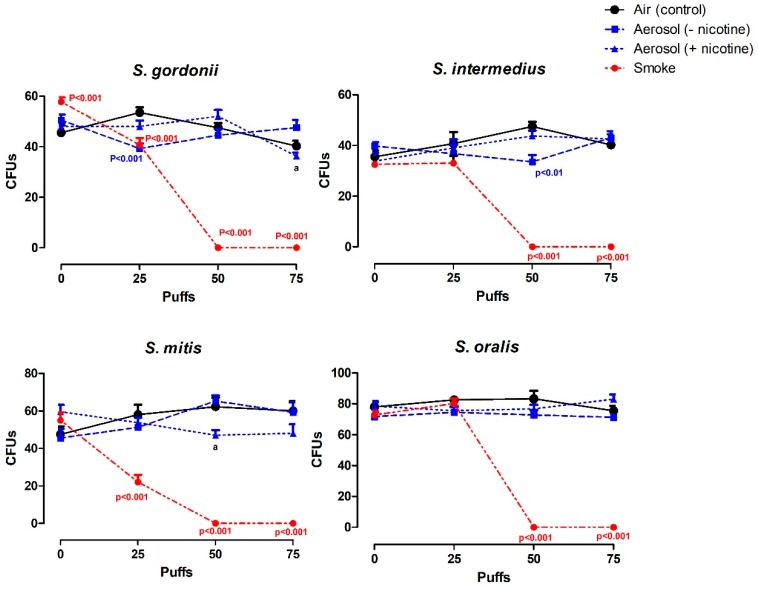
Effect of air, flavorless ECIG aerosol (±20 mg/mL nicotine), or smoke (Marlboro^®^ Red cigarette) on colony-forming unit (CFU) counts. Each data point is Mean ± standard error (SE), *n* = 4 (average of triplicates from each quadrant of an agar plate), p values indicate significance from control. a = *p* < 0.01 between aerosol with nicotine and without nicotine.

CFU counts of commensal oral *streptococci* seeded on agar and exposed to puffs of air (control), flavorless ECIG aerosol ± nicotine and cigarette smoke prior to overnight colony growth are shown in Figure 3. Without exposure (0 puffs), the number of CFUs per agar plate ranged between 37 and 63 for *S. gordonii*, 25 and 42 for *S. intermedius*, 35 and 70 for *S. mitis*, and 65 and 84 for *S. oralis*. Bacteria exposed to flavorless ECIG aerosol ± nicotine grew similar numbers of colonies as compared to those exposed to air, although significant differences (*p* < 0.01) between the aerosol with and aerosol without nicotine exist for *S. gordonii* and *S. mitis* at 75 and 50 puffs, respectively (Figure 3). In drastic contrast, bacteria exposed to 50 or 75 puffs of cigarette smoke yielded no colonies at all. Our results indicate a profound toxic effect of cigarette smoke and a far lower toxic effect of flavorless ECIG aerosol on commensal oral *streptococci*.

### 3.3. Effects of ECIG-Generated Aerosol and Smoke on Colony Size

**Figure 4:**
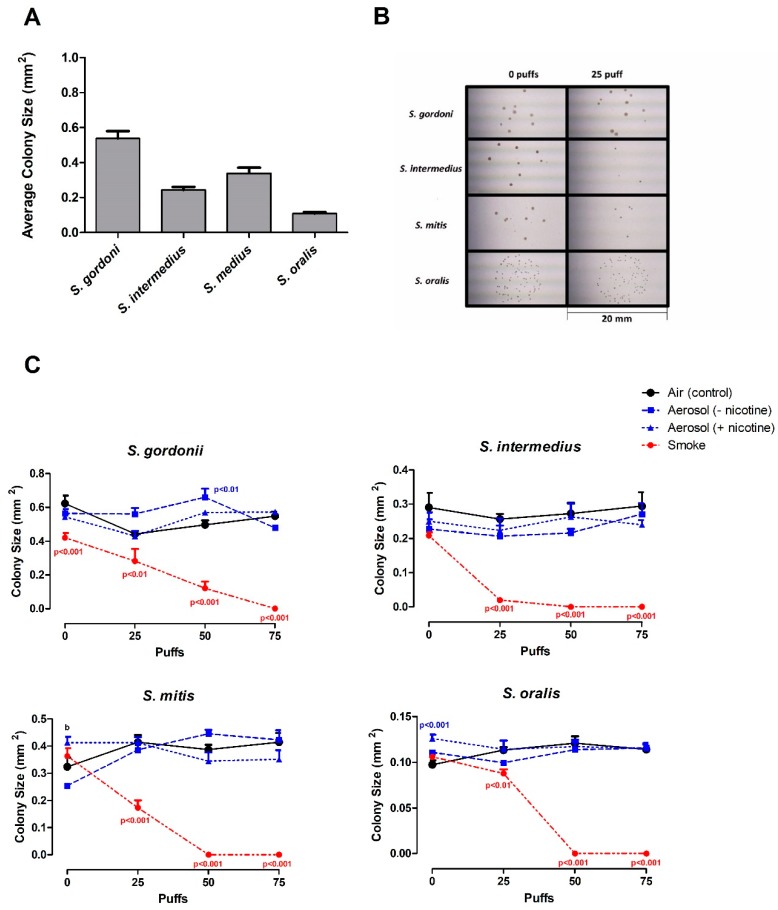
Effect of air, flavorless ECIG aerosol (±20 mg/mL nicotine), or smoke (Marlboro^®^ Red cigarette) on CFU size. **(A)** Average colony size of commensal oral *streptococci*. The average of the three largest colonies in each quadrant of four agar plate (n=16) were used to determine mean colony size for all bacteria at every exposure. Each data point is the Mean ± SE (mm^2^). (**B)** Representative images of colony sizes of commensal oral *streptococci* following exposure of 0 and 25 puffs of smoke (Marlboro^®^ Red cigarette). Each frame is 20 mm long. (**C)** Effect of air (control), aerosol (±20 mg/mL nicotine), or smoke (Marlboro^®^ Red cigarette) on colony size of commensal oral *streptococci*. Each data point is Mean ± SE, *n* = 4, p values indicate significance from control. b = *p* < 0.001 between aerosol with nicotine and without nicotine.

Besides the obvious absence of colonies following exposure to 50 and 75 puffs of smoke, colonies exposed to 25 puffs of cigarette smoke also appear to have a smaller size compared to those colonies exposed to air or flavorless ECIG aerosol ± nicotine. Figure 4A displays the average area of colony sizes for *S. gordonii*, *S. intermedius*, *S. mitis* and *S. oralis* without exposure (0 puffs) which are 0.543 ± 0.023, 0.244 ± 0.015, 0.339 ± 0.018 and 0.110 ± 0.003 mm^2^, respectively. Figure 4B highlights the smaller sizes of colonies for all bacteria after exposure to 25 puffs of smoke compared to colonies after exposure to zero puffs. Since no colonies of *S. intermedius*, *S. mitis* and *S. oralis* were able to grow on agar plates after 50 and 75 puffs of smoke, these are not shown in Figure 4B. However, exposure of *S. gordonnii* to 50 puffs smoke did reduce colony size by 71% as compared to 0 puffs and 57 % as compared to 25 puffs. Colony sizes after exposure to 0, 25, 50 and 75 puffs of air (control), flavorless ECIG aerosol ± nicotine and cigarette smoke are quantified in Figure 4C. The average colony size of all bacteria exposed to 25 puffs of smoke are significantly smaller (*p* < 0.01 to *p* < 0.001) than the controls. Flavorless ECIG aerosol without nicotine also appears to cause a slight increase (*p* < 0.01) on the colony size of *S. gordonii* after exposure to 50 puffs as compared to control (Figure 4C). In all other species tested there are no significant differences among colony sizes after exposure to ECIG aerosol ± nicotine compared to control (Figure 4C).

### 3.4. Effects of ECIG-Generated Aerosol and Smoke on Bacterial Biofilms

**Figure 5:**
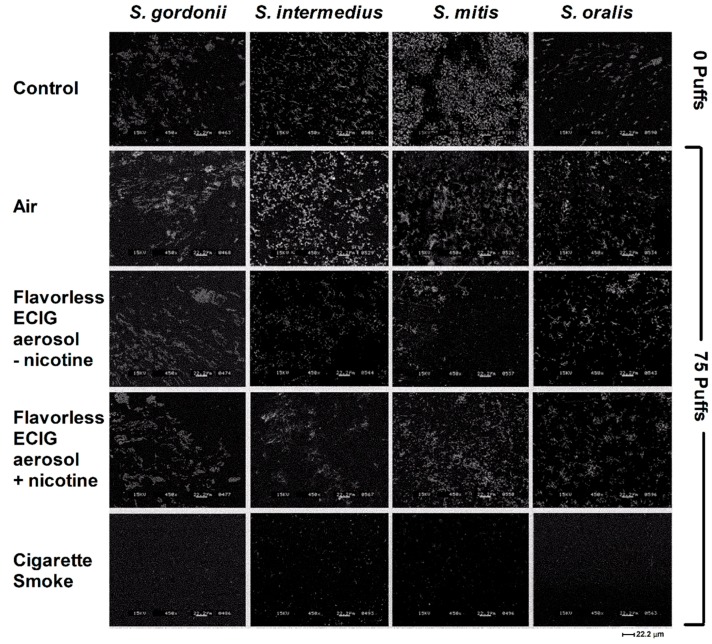
Effect of air, flavorless ECIG aerosol (±20 mg/mL nicotine), or smoke (Marlboro^®^ Red cigarette) on biofilm formation. Representative images of biofilm formation for commensal oral *streptococci* following exposure to 0 puffs of air (control) or 75 puffs of air (control), aerosol (±20 mg/mL nicotine) and smoke (Marlboro^®^ Red cigarette). All images were acquired at 450× using an acceleration voltage of 15 kV.

Figure 5 illustrates the formation of single-species bacterial biofilms grown in 50% BHI (v/v) on plastic coverslips 24 h after exposure to 0 puffs of air (control), 75 puffs of air (control), flavorless ECIG aerosol ± nicotine and cigarette smoke. Concentrations above or below 50% BHI resulted in either mostly planktonic bacteria or not enough biofilm growth, respectively (data not shown). As shown, each of these species is able to grow biofilms after exposure to air (control) and flavorless ECIG aerosol ± nicotine, but not cigarette smoke. Compared to air exposures (0 and 75 puffs), 75 puffs of flavorless ECIG aerosol ± nicotine is permissive for biofilm formation and growth regardless of the overall architecture of bacterial communities for all four species. Although debatable, exposure to ECIG aerosol ± nicotine may have yielded a lower overall amount of biofilm growth in all species when compared to air exposure. Since Figure 5 is merely qualitative data, it is hard to draw strong conclusions about this point. Notwithstanding this, it is also obvious that such treatments still allow for biofilm growth. These results indicate that flavorless ECIG aerosol ± nicotine has at most a modest effect on oral commensal *streptococci* biofilm formation and growth under these conditions.

## 4. Discussion

The current work demonstrates that flavorless ECIG aerosol ± nicotine has little to no toxic effect on the in vitro growth of the four oral commensal *streptococci* tested here. Our data show that CFUs for all four species exposed to flavorless ECIG aerosol ± nicotine can grow to similar numbers and to similar sizes as compared to their untreated counterparts. Our data also demonstrate that bacteria attached to coverslips and exposed to flavorless ECIG aerosol ± nicotine are also able to grow biofilms like their untreated controls. However, when bacteria are exposed to cigarette smoke, the growth of colonies and biofilms is severely impaired or completely obliterated. Furthermore, it is evident that nicotine is not the culprit of this impairment since ECIG aerosol contained a higher concentration of nicotine than cigarette smoke on a per puff basis (Figure 2). Based on these results, we propose that flavorless ECIG aerosol ± nicotine does no apparent harm to these four oral bacteria under the conditions tested. However, it remains to be seen what effect the addition of various flavors to the E-liquid would have on the growth of these streptococcal bacteria; work which is currently underway in our laboratory.

While the CFU data and the colony size data indicate that aerosol (± nicotine) has little or no effect on bacterial survival and growth, it could also be argued that biofilm formation is somewhat dampened when bacteria (particularly *S. mitis*) are exposed to aerosol without nicotine. Furthermore, this effect appears to be reversed when nicotine is added to the aerosol. If true, this would mean that the base humectants (i.e., vaporized propylene glycol and glycerol) may have an inhibitory effect on bacterial survival and growth while nicotine (at the dose tested) has a stimulatory effect on bacterial survival and growth. However, without further experimental evidence, we cannot state definitely that this is actually the case. Consequently, our laboratory is currently investigating the effects of bubbling aerosol (± nicotine) into BHI broth before allowing for planktonic growth of these streptococcal bacteria.

Overall, flavorless ECIG aerosol (±nicotine) appears to have little or no effect on CFU number and colony size of all bacteria tested (Figure 3 and Figure 4). *S. gordonii* and *S. intermedius* exhibit a slight decrease in CFU numbers when exposed to 25 and 50 puffs of aerosol without nicotine, respectively, and *S. gordonii* exhibits a slight increase in colony size when exposed to 50 puffs of aerosol without nicotine. Furthermore, the presence or absence of nicotine in the flavorless ECIG aerosol also appears to have little effect on CFU number and colony size of all *streptococci* tested. One may argue that such differences are solely based on the treatments and, in fact, ECIG aerosol (±nicotine) does have a slight but significant effect on the growth of these oral bacteria. However, due to the small changes seen, one may also argue that these differences are due to inherent variability associated with CFU plating [41]. On a related subject, it is also worth noting that this study does not address the number of bacteria within each colony. Therefore, it is possible to find different bacterial densities before and after treatments. A modest indication of low variability in bacterial density is seen in Figure 5 where we can observe the overall effect of treatments on the density of micro-colonies. The results of this investigation are comparable to the results of Huang et al. (2014) who report no significant difference in planktonic growth of *S. gordonii* in trypticase soy broth growth media or of CFU counts on tryptic soy agar plates at nicotine concentrations below 1 mg/mL [31], although nicotine concentrations between 1 and 4 mg/mL appeared to stimulate *S. gordonii* planktonic growth in a dose-dependent manner. It is most likely that the effect of nicotine on streptococcal bacteria is species-dependent, since Li et al. (2014) determined that nicotine had little effect on *S. sanguinis* biofilm formation, but increased *S. mutans* biofilm formation [30].

Oral bacteria live in polymicrobial communities [42], even when exposed to cigarette smoke. The study by Shah et al. (2017) indicates that, in polymicrobial biofilms, commensal species typically suffer and struggle to grow in the presence of tobacco smoke while pathogenic species thrive under the same conditions [43]. The decrease in growth of commensal bacteria exposed to cigarette smoke (Figure 3, Figure 4 and Figure 5) may be due to down regulation of important metabolic factors in the commensal species [43]. Interestingly, Zonuz et al. (2008) reported accelerated growth of *S. sanguinis* and *S. mutans* in the vicinity of cigarette smoke [44]—an apparently contradictory finding. The findings of the present study are in agreement with Shah et al. (2017), in which commensal bacteria are unable to form single-species biofilms when exposed to cigarette smoke. Assuming the microbial landscape within the oral cavity shifts toward poor oral health in response to cigarette smoke, as Shah et al. (2017) indicate, this could ultimately lead to more severe pathophysiologic problems [43]. For example, the report by Bagaitkar et al. (2011) suggests that cigarette smoke extract augments the persistence of *P. gingivalis* in biofilms with *S. gordonii* by elevated expression of major fimbrial antigen [33]. *P. gingivalis* is a Gram-negative pathogenic bacterium and a principal periodontitis-inducing agent [45,46]. Periodontal pathogens induce systemic inflammation, ultimately leading to increased risk of cardiovascular disease such as atherosclerosis [47,48]. While flavorless ECIG aerosol has no effect on our commensal bacteria’s ability to form biofilms (Figure 5), it is important to test the effects of conventional cigarette smoke and ECIG aerosol with mixed-species biofilms to determine the effects of the same environmental agents in an open system. The results of such experiments will give a much better understanding of the effects of smoke and ECIG aerosol on oral microbial communities. Our experimental design also tested only one strain of bacteria at a time. Interactions between strains of commensal bacteria, as well as with pathogenic bacteria, should be investigated to determine ultimately if the effect of flavorless ECIG-aerosol is also different than that of smoke in mixed species biofilms. To best address this question, the bacteria should be cultured in an open system following exposure. An open system design would resemble the natural oral environment in the context of mixtures of oral microbiota combined with salivary flow, which aids in clearance of external materials from the oral cavity.

As CFU counts, colony size and biofilm formation obtained from this investigation indicate, we are confident that flavorless ECIG aerosol has a less drastic effect on the oral commensal bacteria tested than cigarette smoke. However, this study does have limitations. The in-house prepared E-liquid used in this study represents a single rendition of E-liquid and does not represent the flavored preferences of most ECIG users, particularly high-school teens who are enticed by the many exotic flavors offered by ECIG companies. In addition, the only concentration of nicotine used is 20 mg/mL and the only ratio of propylene glycol to vegetable glycerin is 1:1 *v/v*. Furthermore, the only bacteria tested were these four commensal *streptococci*, which were evaluated individually. Red complex bacteria, such as *P. gingivalis*, *Aggregatibacter actinomycetemcomitans*, *Tannerella forsythia* or *Treponema denticola* were not included in this study, presenting another important limitation. Therefore, conclusions concerning the effects of ECIG aerosol on the survival and growth of commensal bacteria must be considered within the context of variability in the composition of commercially available E-liquids and the bacteria evaluated. Another limitation is the fact that flavorless ECIG-aerosol and conventional cigarette smoke are not identical by nature [49]. For example, the E-liquid vaporization process versus the tobacco combustion process result in exposure chambers with different physical environments such as temperature and humidity [40]. Furthermore, the volume of the human oral cavity, as determined by height, width and depth [50], is approximately 230 cm^3^, much smaller than the 2100 cm^3^ calculated for the exposure chambers used in this study [40]. This means that the effect of flavorless ECIG-aerosol or conventional cigarette smoke on oral commensal *streptococci* could be minimized in this investigation as compared to their effects in vivo. Alternatively, the in vitro results of this study could also be amplified when comparing the effect of aerosol and smoke on oral commensal *streptococci*. For example, our results clearly show that the effects of 50 puffs of conventional smoke strongly obliterates colony or biofilm formation in every oral species tested. However, it is important to note that our in vitro experiments do not have a mechanism for removal of aerosol or smoke materials as saliva would in the oral cavity in vivo, but rather are constantly exposed to these materials for the duration of the experiments. In addition to lacking removal mechanisms of aerosol or smoke material, the slow buildup of aerosol and especially smoke within the chamber very likely changes the environment within the chamber to be less oxygenated, a condition which undoubtedly affects the growth of even facultative bacteria. Consequently, the results obtained from using this exposure system may (in part) be due to decreased oxygenation.

Moreover, since our experimental design does not exhibit the properties of an open system, it cannot determine whether the overall effect of cigarette smoke on oral commensal bacteria is bacteriostatic or bactericidal. In the scenario where the smoke is bacteriostatic, such experimental design will allow for the removal of potential bacteriostatic compounds and once the cigarette smoke materials fall below the minimal inhibitory concentration, the bacterial communities will be able to resume growth. It is important to note that the growth and/or architecture of biofilm communities may be altered at this point as a result of such cigarette smoke effects. Alternatively, if the smoke materials are bactericidal, the bacteria will be dead even after the removal of such materials. This aspect deserves further study because the livelihood of the commensal bacteria is important to the homeostasis of the oral cavity, keeping it and the entire physiological system healthy. Consequently, we are currently in the process of developing an open system design to address these questions.

## 5. Conclusions

This study indicates that flavorless ECIG aerosol (±nicotine) is less detrimental to the survival and growth of oral commensal *streptococci* than conventional cigarette smoke. This study opens the door for subsequent studies that could address the effect of flavorless ECIG aerosol on oral epithelial cells as well as the addition of flavoring agents to test all the aforementioned biological models.

## Figures and Tables

**Table 1:** Pump/chamber parameters and nicotine concentrations within the exposure chambers.

	Smoke Pump/Chamber	Aerosol Pump/Chamber
**Pump Flow Rate (mL/min)**	400	400
**Puff Duration (s)**	5	5
**Puff Volume (mL)**	33.3	33.3
**Nicotine (mg/cigarette) ^a^**	0.92	2.80
**Nicotine (mg/puff) ^b^**	0.06	0.19
**Nicotine Flow (µg/puff/mL) into Chamber**	1.8	5.7
**Exposure Chamber Volume (cm^3^)**	2126	2126
**Nicotine Delivered to Chamber (mg)**		
0 puffs (0.0 cigarettes)	0.0	0.0
25 puffs (1.7 cigarettes)	1.5	4.8
50 puffs (3.3 cigarettes)	3.0	9.5
75 puffs (5.0 cigarettes)	4.5	14.3
**Nicotine Concentration in Chamber (µg/cm^3^) ^c^**		
0 puffs (0.0 cigarettes)	0.0	0.0
25 puffs (1.7 cigarettes)	0.7	2.2
50 puffs (3.3 cigarettes)	1.4	4.5
75 puffs (5.0 cigarettes)	2.1	6.7

^a^ For smoke, the value of mg nicotine per Marlboro^®^ Red cigarette [35]. For aerosol, the value 2.8 mg/cigarette is based on 9.3 µL of E-liquid (20 mg/mL nicotine) aerosolized per puff and that 15 puffs is equivalent to one cigarette [40]. ^b^ One cigarette is equivalent to 15 puffs. ^c^ These values assume that all nicotine remains within the chamber, which is not the case since the exposure chambers are fitted with rubber end caps perforated with small holes to allow venting of the exposure chambers [36,37].
